# Determinants of dietary patterns of Ghanaian mother-child dyads: A Demographic and Health Survey

**DOI:** 10.1371/journal.pone.0294309

**Published:** 2023-11-14

**Authors:** Clement Kubreziga Kubuga, Dayeon Shin, Won O. Song

**Affiliations:** 1 Nutritional Sciences Department, University for Development Studies, Tamale, Ghana; 2 Department of Food and Nutrition, Inha University, Incheon, Republic of Korea; 3 Food Science and Human Nutrition Department, Michigan State University, EL, MI, United States of America; Wroclaw University of Environmental and Life Sciences: Uniwersytet Przyrodniczy we Wroclawiu, POLAND

## Abstract

Having a comprehensive understanding of a population’s dietary patterns is a key component of any effective strategy for preventing malnutrition, planning, and putting nutrition interventions and policies into place. It’s interesting to note that information on dietary patterns of Ghana’s vulnerable subpopulations of women and children is lacking. The purpose of this study is to characterize the dietary patterns of women (15–49 years old) and their young children (0–3 years old), as well as to investigate into the socioeconomic and demographic factors influencing the characterized dietary patterns. The sociodemographic information and food consumption of mother-child dyads (n = 1,548) were collected for this nationally representative cross-sectional study. Principal component analysis and multiple variable logistic regression were used, respectively, to determine the dietary patterns of dyads and the determinants of the identified dietary patterns. For women and children, respectively, four dietary patterns (‘*Beverage & sugary based’*, *‘Meat based’*, *‘Indigenous- tuber based’ and ‘Indigenous- grain based’*) and two (‘*Indigenous’ and ‘Milk*, *Meat*, *& cereal based’)* emerged. Ethnicity, wealth quintiles, parity, seasonality, dyad’s age, body mass index, education, residency, marital status, and household size were the socioeconomic / demographic determinants of the dietary patterns. To sum up for women and children, meat based and indigenous staple based dietary patterns were identified, with several important socioeconomic and demographic variables acting as predictors of the dietary patterns. The identified dietary patterns and their determinants may serve as a basis for nutrition intervention and policies for women and children in Ghana.

## Introduction

Globally, micronutrients deficiencies are most common in women of reproductive age and children due to increased needs of the subgroups and poor quality of diets [1–4]. In Ghana, undernutrition specifically micronutrients deficiencies are a major problem [5–10] especially among women of reproductive age and children. Among multifaceted causes of micronutrients deficiencies, dietary intake plays a key role [11–13]. Research suggests that an efficient approach to tackling malnutrition and nutrient deficiencies in relation to food intake requires the use of dietary patterns (DP) [14]. DP is said to be "the quantities, proportions, variety, *or combination of different foods*, drinks, and nutrients (when available) in diets, and the frequency with which they are habitually consumed [15]".

DP has been proposed as a method to predict the relationship between diet and development of diseases [16] or health conditions associated with dietary habits. The reasoning behind the suggestion is that foods are frequently taken in combination; it is challenging to distinguish between the influence of individual foods on the development of diseases in observational studies [16, 17]. Interestingly, dietary patterns of the Ghanaian women and their children have not been previously characterized at the national level, this study aimed to utilize the 2008 Ghana Demographic and Health Survey (GDHS) data set to characterize DP of women and their children. Additionally, this study seeks to investigate the socio-economic/demographic factors predicting the characterized DP. The 2008 GDHS data set is used in this study because it currently represents the most recent national data set with food intake for mother-child dyads that is publicly accessible: GDHS data from 2008 includes information on mother-child dyads’ 24-hour food recall intake, but data from 2014 only includes information for children. The 2022 data set is not currently accessible to the public.

**Fig 1:**
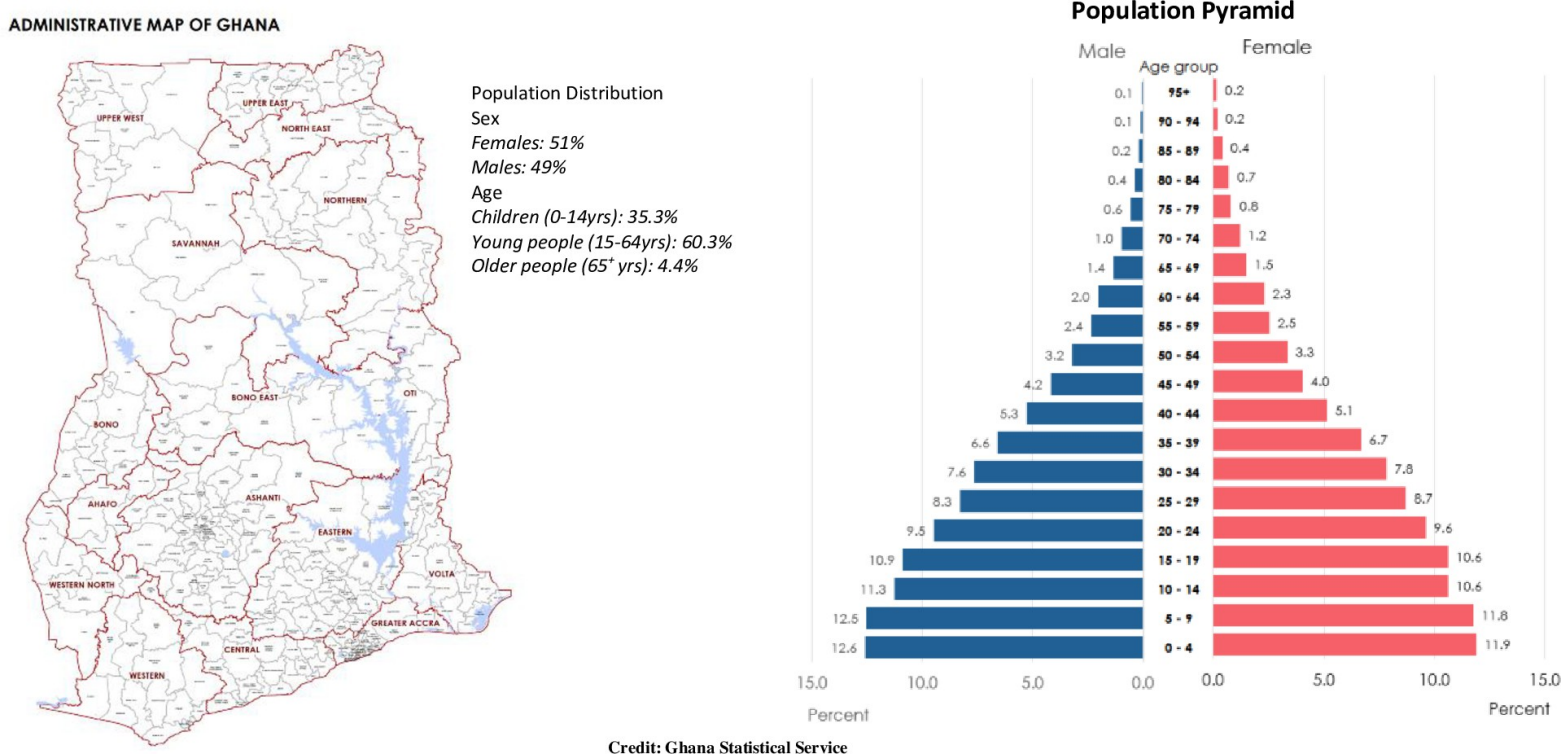
Administrative map of Ghana and population distribution.

Ghana’s population is predominantly young and female dominated (females: 51%; Males: 49%), with about 35% being children, 60% being young people, and about 4% being in the older population (Fig 1) [18]. This predominantly youthful and female dominated population is saddled with micronutrient deficiencies [19, 20]. Ghana’s vulnerable sub population of women and children is the hardest hit with these micronutrient deficiencies [19, 20]. Though dietary patterns are known to predict such deficiencies [16], dietary patterns of women and their children in Ghana are yet to be characterized. To contribute to optimal nutrition for women and their children in Ghana, characterization of their dietary patterns is urgently needed. It is well known that maternal and early life nutrition of children predict adulthood nutritional status, intergenerational nutrition and wellbeing [21]. Optimal nutrition in this sub group (mothers and their children) would invariably translate to good nutrition in the next generation [22]. As such, the survival of every nation is dependent on its children’s nutritional status and their mothers’ which are tied to their dietary patterns.

Dietary patterns have been derived by various approaches such as reduced rank regression, principal component analysis and factor analysis [23–27]. Commonly dietary patterns have been derived by reduced rank regression [23], dietary intake by index analysis [28] and factor analysis [29] or individual nutrient intakes [30]. The approach applied is largely based on the focus of the study and the nature of data. We employed principal component analysis in this study based on the qualitative nature of the food intake data.

## Materials and methods

### Study design and subjects

This study examined data from the 2008 Ghana Demographic Health Survey (GDHS), a cross-sectional nationwide study that included data from every region of the nation. The GDHS is normally carried out every five years. The five-year period creates a balance between gathering timely data and giving adequate time for demographic and health indicators to undergo significant changes. It enables the development of trustworthy data for the execution of programs and evidence-based policymaking. GDHS collects information on a variety of topics, including households and housing characteristics, education, nutrition, maternal and child health, family planning, gender, domestic violence, and knowledge and behavior about HIV/AIDS [9]. The 2008 GDHS data set is used in this study because, as was mentioned earlier, it is the most recent national data set that includes food intake for mother-child dyads and is available to the general public. Additionally, the findings of this study could serve as a crucial basis for comparative and trend analysis of dietary patterns for Ghanaian mother-child dyad in the future. In the aforementioned data set, information for a total of 4,916 women between the ages of 15 and 49 was obtained. Also, information on children under five years in selected households was gathered. Mothers who had children under the age of three and lived together were interviewed about their food intake [9]. Details about the methodology and design of the GDHS has been published by the Ghana Statistical Service, Ghana Health Service, and ICF Macro [9].

### Mother-child dyad food consumption assessment

Mother-child dyad food consumption assessment is very crucial in contributing to mother-child dyad’s optimal nutrition. Ghana is saddled with micronutrient deficiency prevalence which greatly affects the vulnerable sub population of women and children [19, 20] with the nation’s health authorities faced with the task of unraveling the causes and provision of appropriate interventions. Though dietary patterns are known to predict such deficiencies [16], dietary patterns of the Ghanaian women and their children are yet to be characterized at the national level, this work seeks to contribute to filling this gap.

In this current work, information on all women (15–49 years old, n = 4,916) in the 2008 GDHS and their children was extracted. Further extraction of data on mothers and their children (0–3 years) with 24hr recall food intake data, resulted in 1,548 mother-child dyads in this category. In the 2008 GDHS, only mothers who had children under the age of three and lived together were interviewed about their food intake. Based on their replies (’yes’ for consumption or ’no’ for non-consumption’) to a predetermined list of food items grouped per the Demographic and Health Survey program’s classification, qualitative responses on mother-child pairs were collected (Table 1). It is worth mentioning that we were excited to showcase some preliminary observations of this study at a conference as a published abstract (which featured in journal Current Developments in Nutrition) [31].

**Table 1:** Number and proportion of mother-child dyads consuming different food items on the day and night preceding demographic and health survey interview date by current breastfeeding and pregnancy status: The 2008 Ghana Demographic and Health Surveys.

Food group consumed on day and night preceding the interview	Mothers (n = 1,548)						Children (n = 1,548)		
	Breastfeeding			Pregnant			Being breastfed		
	yes (n = 1,112)	no (n = 436)	p-value	yes (n = 106)	no (n = 1,442)	p-value	yes (n = 1,112	no (n = 436)	p-value
	n (%)	n (%)		n (%)	n (%)		n (%)	n (%)	
Beans, peas, lentils, legumes and nuts	314(28.4)	118(27.3)	0.655	26(24.5)	406(28.3)	0.400	180(16.3)	119(27.4)	<0.000
Bread, noodles or other grains/cereals foods	965(87.0)	377(87.5)	0.811	94(88.7)	1248(87.0)	0.624	611(55.2)	390(89.9)	<0.000
Cheese, yogurt or other milk products	79(7.2)	32(7.4)	0.852	6(5.7)	105(7.3)	0.519	57(5.2)	49(11.4)	<0.000
Chocolates, sweets, candies,	170(15.4)	74(17.2)	0.390	19(17.9)	225(15.7)	0.552	229(20.7)	200(46.1)	<0.000
Dark green leafy vegetable	637(57.4)	220(50.7)	0.017	57(53.8)	800(55.6)	0.710	342(30.9)	215(49.5)	<0.000
fish or shellfish (fresh or dry)	799(72.2)	318(73.6)	0.588	77(72.6)	1040(72.6)	0.997	425(38.4)	313(72.5)	<0.000
Liver, heart or other inside organs of meats	120(10.8)	41(9.5)	0.429	12(11.3)	149(10.4)	0.763	59(5.3)	44(10.1)	0.001
Mangoes, papayas or other vitamin A-based fruits	101(9.1)	46(10.6)	0.362	13(12.4)	134(9.3)	0.303	54(4.9)	58913.4)	<0.000
Meats (beef, pork, lamb, chicken)	317(28.7)	138(31.8)	0.225	36(34.0)	419(29.2)	0.302	125(11.3)	130(30.0)	<0.000
Eggs	206(18.6)	87(20.2)	0.470	18(17.1)	275(19.2)	0.611	153(13.8)	139(32.0)	<0.000
Oil, fats, butter, products made from them	568(51.3)	245(56.7)	0.054	57(54.3)	756(52.7)	0.751	310(28.1)	258(59.7)	<0.000
Other fruit	713(64.2)	273(63.2)	0.703	57(54.8)	929(64.6)	0.045	403(36.4)	284(65.4)	<0.000
Pumpkins, carrots, yellow or orange squash	211(19.0)	65(15.0)	0.062	19(17.9)	257(17.9)	0.992	106(9.6)	60(13.8)	0.016
Potatoes, cassava or other tubers	704(63.4)	279(64.4)	0.695	69(65.1)	914(63.6)	0.751	328(29.6)	267(61.5)	<0.000
Tea or coffee	240(21.7)	82(18.9)	0.225	18(17.1)	304(21.2)	0.321	112(10.1)	93(21.5)	<0.000
Tinned/powder or fresh milk	158(14.3)	85(19.7)	0.010	20(18.9)	223(15.6)	0.375	149(13.5)	114(26.3)	<0.000
Other solid or semi-solid foods	-	-	-	-	-	-	272(24.6)	147(34.1)	0.000
Baby formula	-	-	-	-	-	-	68(6.2)	24(5.6)	0.654
Baby cereal	-	-	-	-	-	-	86(7.8)	44(10.2)	0.130
Other porridge/gruel	-	-	-	-	-	-	494(44.5)	266(61.4)	<0.000

*P-values based on the Pearson chi-square test.

### Statistical analysis

Data analyses were carried out using SAS 9.4 (SAS Institute Inc., Cary, NC, USA). Distribution of mother-child dyad food consumption per the 24 hr recall by current pregnant and breastfeeding status was examined by Pearson chi-square test. Dietary patterns of the mother-child dyads were identified by subjecting food items reported in Table 1 to principal component analysis (PCA) as the extraction procedure. The data variation is explained by principal components, which are linear combinations of the input variables. The linear combination allows for the computation of a component score for each woman/child, and each component specifies a dietary pattern. Several techniques (parallel analysis, visual scree test and literature search) were employed to determine the total number of factors for retention. The understanding of components was aided by the application of varimax rotation. Factor sufficiency was determined a priori, and pattern coefficients ≥ ±0.40 were deemed salient and practically relevant. In the goal of parsimony and to be consistent with simple structure, complex loadings salient on multiple factors were discarded. Components loaded with a single variable were likewise rejected. Factors with internal consistency and at least three salient pattern coefficients. A factor was deemed adequate if it had at least three salient pattern coefficients and an internal consistency reliability ≥ 0.70. Using factor loadings with a Cochran’s alpha level ≥0.60, internal reliability was examined.

Multivariable logistic regression models were used to examine the relationship between socio-economic/ demographic determinants of mother-child dyads and dietary patterns. All the studied covariates were simultaneously adjusted in a multiple regression model. we modelled the probability of being in the upper tertile of each dietary pattern as the outcome of interest. In the interest of simplicity, patterns observed across the whole research population were used to identify the determinants of dietary patterns. Nonetheless, sub strata were taken into account in all models (current breastfeeding and pregnancy status). Number of household members, parity, month of interview, maternal age, maternal body mass index (BMI), religion, ethnicity, marital status, number of household members, place of residence, region of residence, maternal education, wealth index, household head sex and age, type of union, maternal alcohol consumption, and number of children under five years old were among the key covariates used. The children’s models also included the child’s age and sex. The cutoff for statistical significance was P < 0.05. The results are presented as the coefficients of the variables and their accompanying p-values. Individuals whose data were missing were not included in the analysis.

## Results

### Mother-child dyads characteristics and food consumption

Table 2 (second column) indicates the distribution of the characteristics of the mothers. Key highlights of essence is that most households (46%) had a membership of 6, more of the households (73%) had male household heads. Majority of the women (72%) were married or living together with their partners, majority of the women (66%) lived in rural areas. 20% of the women consume alcohol and a small fraction of the women (7%) were currently pregnant. Half of the children (50%) were males, most of the children (35%) were in the age brackets of 13–24 months (Table 3, second column).

**Table 2:** Determinants of dietary patterns of Ghanaian mothers: The 2008 Ghana Demographic and Health Survey.

Parameter	*Likelihood of being on the upper tertile of the dietary patterns (α = 0.05)								
		Beverage & sugary based		Meat based		Indigenous- tuber based		Indigenous- grain based	
	n(%)	estimate	P-value	estimate	P-value	estimate	P-value	estimate	P-value
**Number of household members**									
2 members	59(4)	0.0	1.000	-0.1	0.721	-0.7	0.110	-0.3	0.469
3 members	242(17)	0.2	0.448	-0.1	0.578	-0.3	0.299	-0.1	0.590
4 members	272(18)	0.0	0.836	-0.1	0.495	0.1	0.618	0.2	0.452
5 members*	265(17)	0.5	0.013	-0.3	0.109	0.0	0.953	0.1	0.516
6 members	710(46)				Ref				
**Marital status**									
Married/living together*	1120(72)	0.7	0.048	0.2	0.640	0.0	0.983	-0.1	0.864
Divorced/Separated	278(18)	0.6	0.133	-0.2	0.631	-0.2	0.510	-0.4	0.258
Widowed	10(1)	1.6	0.133	0.1	0.934	0.6	0.512	-2.2	0.050
Never married/lived together	142(9)				Ref				
**Body mass index (BMI)**									
Underweight	135(9)	-0.3	0.351	-0.6	0.072	-0.2	0.659	-0.5	0.138
Normal weight	1022(66)	-0.3	0.303	-0.5	0.073	0.2	0.371	0.0	0.846
Overweight	283(18)	0.0	0.990	-0.5	0.054	-0.1	0.777	-0.5	0.068
Obese	97(8)				Ref				
**Wealth quintile**									
Lowest*	478(31)	-1.7	< .000	0.0	0.907	0.0	0.925	-0.4	0.235
Second*	347(22)	-1.4	< .000	-0.4	0.198	-0.1	0.728	0.1	0.780
Middle*	260(17)	-0.7	0.011	-0.4	0.093	0.2	0.393	-0.2	0.550
Fourth*	275(18)	-0.8	0.001	0.0	0.985	0.0	0.936	-0.1	0.525
Highest	188(12)				Ref				
**Household head sex**									
Male	1137(73)	-0.1	0.610	-0.2	0.150	0.0	0.976	-0.1	0.632
Female	411(27)				Ref				
**Age of household head**									
15–25*	160(10)	0.2	0.496	0.1	0.782	0.4	0.132	-0.6	0.029
26–35	534(35)	0.1	0.531	0.0	0.880	0.0	0.939	-0.2	0.238
36^+^	854(55)				Ref				
**Kind of union**									
Monogamy	1146(79)	0.1	0.695	-0.3	0.108	-0.2	0.223	-0.2	0.265
Polygamy	309(21)				Ref				
**Highest education attained**									
No education	377(38)	-0.5	0.341	-0.4	0.335	-0.1	0.845	-0.8	0.055
Primary	477(48)	-0.2	0.607	-0.3	0.420	0.1	0.748	-0.7	0.075
Middle/JSS	101(10)	-0.1	0.889	-0.5	0.216	0.2	0.616	-0.9	0.025
Secondary and above	36(4)				Ref				
**Number of children (<5yrs)**									
0	6(0.4)	-0.7	0.445	0.0	0.985	-0.9	0.393	-1.6	0.196
1	668(43)	-0.5	0.321	0.3	0.530	0.9	0.060	0.5	0.318
2	638(41)	-0.4	0.370	0.5	0.265	0.7	0.167	0.5	0.322
3	176(11)	-0.4	0.386	0.3	0.561	0.9	0.081	0.6	0.201
4	60(4)				Ref				
**Pregnancy status**									
Not pregnant	1442(93)	0.1	0.852	0.0	0.936	0.2	0.365	0.1	0.617
Pregnant	106(7)				Ref				
**Religion**									
Christians	1046(71)	0.1	0.865	-0.5	0.275	0.3	0.510	0.6	0.199
Islam	313(21)	0.4	0.415	-1.0	0.067	0.1	0.778	0.2	0.687
Traditionalist/other	115(8)				Ref				
**Ethnicity**									
Akan	597(39)	0.5	0.324	-0.9	0.082	1.2	0.018*	-1.1	0.023*
Ga/Dangme	69(4)	0.8	0.172	-0.9	0.098	0.6	0.312	-0.8	0.120
Ewe	203(13)	1.2	0.025*	-1.3	0.016*	1.2	0.027*	-0.3	0.522
Guan	42(3)	0.1	0.846	-0.8	0.236	1.5	0.032*	-0.7	0.301
Mole-Dagbani	387(25)	1.1	0.059	-1.2	0.024*	0.6	0.260	-0.7	0.190
Grussi	93(6)	0.8	0.208	-0.9	0.146	0.4	0.482	0.0	0.943
Gruma	94(6)	0.7	0.356	0.1	0.935	0.8	0.291	-0.8	0.263
Other	62(4)				Ref				
**Region of residence**									
Southern Ghana	1033(67)	-0.3	0.401	-0.5	0.096	0.1	0.729	-1.0	0.001*
Northern Ghana	515(33)				Ref				
**Place of residence**									
Urban	526(34)	0.3	0.063	0.0	0.788	-0.2	0.269	0.1	0.683
Rural	1022(66)				Ref				
**Age of women**									
15–25	571(37)	-0.3	0.264	0.2	0.570	-0.5	0.076	-0.2	0.409
26–35	688(44)	0.1	0.573	0.3	0.130	-0.4	0.051	-0.2	0.486
36–49	289(19)				Ref				
**Month of interview**									
September	610(39)	0.6	0.004*	0.0	0.810	-0.1	0.458	-0.3	0.143
October	618(40)	0.1	0.729	-0.1	0.722	-0.3	0.170	0.0	0.840
November	320(21)				Ref				
**Consumes alcohol**									
No	1232(80)	0.0	0.909	-0.1	0.759	0.0	0.932	-0.1	0.564
Yes	315(20)				Ref				
**Number of children ever born**									
1 child	338(22)	0.9	0.002*	0.6	0.021*	0.1	0.795	0.3	0.274
2 children	341(22)	0.4	0.09	0.2	0.332	-0.1	0.731	0.0	0.891
3 children	276(18)	0.2	0.307	0.1	0.593	0.2	0.443	0.0	0.877
4 children	593(38)				Ref				

**Table 3:** Determinants of dietary patterns of Ghanaian children: The 2008 Ghana Demographic and Health Survey.

Parameter	*Likelihood of being on the upper tertile of the dietary patterns (α = 0.05)				
		*Indigenous*		*Milk*, *Meat*, *& cereal based*	
	n(%)	estimate	P-value	estimate	P-value
**Sex of child**					
Male	778(50)	0.3	0.088	0.0	0.977
Female	770(50)		Ref		
**Age of child in months**										
≤6	364(24)	-5.4	< .0001	-0.3	0.185
7–12	312(20)	-1.9	< .0001	0.0	0.841
13–24	534(35)	-0.3	0.138	-0.1	0.606
25–35	330(21)		Ref		
**Age of mother**										
15–25	571(37)	-0.5	0.106	0.0	0.868
26–35	688(44)	-0.7	0.011	0.4	0.109
36–49	289(19)		Ref		
**Marital status**					
Married/living together	1120(72)	-0.2	0.693	0.3	0.441
Divorced/Separated	278(18)	-0.1	0.782	0.2	0.604
Widowed	10(1)	0.2	0.880	1.7	0.119
Never married/lived together	142(9)		Ref		
**Mother body mass index**					
Underweight	135(9)	0.0	0.997	-0.7	0.040
Normal weight	1022(66)	0.5	0.117	-0.6	0.018
Overweight	283(18)	0.1	0.650	-0.3	0.337
Obese	97(8)		Ref		
**Wealth quintile**					
Lowest	478(31)	0.3	0.449	-1.7	< .0001
Second	347(22)	0.0	0.913	-1.2	< .0001
Middle	260(17)	0.2	0.618	-0.9	0.001
Fourth	275(18)	0.3	0.224	-0.7	0.005
Highest	188(12)				
**Household head sex**					
Male	1137(73)	0.1	0.784	0.0	0.896
Female	41127)		Ref		
**Age of household head**					
15–25	160(10)	0.2	0.594	-0.1	0.770
26–35	534(35)	0.2	0.316	-0.2	0.208
36^+^	854(55)		Ref		
**Kind of union**					
Monogamy	1146(79)	-0.1	0.602	0.0	0.904
Polygamy	309(21)		Ref		
**Highest education mother attained**										
No education	377(38)	0.7	0.124	-0.6	0.251
Primary	477(48)	0.9	0.047	-0.3	0.523
Middle/JSS	101(10)	0.5	0.300	-0.2	0.629
Secondary and above	36(4)		Ref		
**Number of household members**										
2 members	59(4)	0.3	0.481	-0.3	0.439
3 members	242(17)	-0.2	0.484	0.2	0.429
4 members	272(18)	0.2	0.413	-0.1	0.582
5 members	265(17)	0.1	0.651	0.1	0.507
6 members	710(46)		Ref		
**number of children (<5yrs)**										
0	6(0.4)	-1.2	0.335	-2.9	0.016
1	668(43)	0.0	0.950	-0.7	0.130
2	638(41)	-0.2	0.754	-0.7	0.147
3	176(11)	0.2	0.722	-0.7	0.137
4	60(4)		Ref		
**Pregnancy status**					
Not pregnant	1442(93)	-0.1	0.833	0.4	0.125
Pregnant	106(7)		Ref		
**Religion**					
Christians	1046(71)	0.3	0.522	0.0	0.983
Islam	313(21)	0.0	0.952	0.1	0.923
Traditionalist/other	115(8)		Ref		
**Ethnicity**					
Akan	597(39)	-0.1	0.818	-0.5	0.323
Ga/Dangme	69(4)	0.3	0.660	-0.2	0.763
Ewe	203(13)	0.4	0.455	-0.4	0.479
Guan	42(3)	-0.5	0.527	-0.7	0.312
Mole-Dagbani	387(25)	0.1	0.811	-0.5	0.377
Grussi	93(6)	-0.8	0.274	-1.0	0.098
Gruma	94(6)	0.0	0.976	0.2	0.750
Other	62(4)		Ref		
**Region of residence**					
Southern Ghana	1033(67)	-0.5	0.151	-0.1	0.862
Northern Ghana	515(33)		Ref		
**Place of residence**					
Urban	526(34)	-0.1	0.555	0.1	0.727
Rural	1022(66)		Ref		
**Month of interview**					
September	610(39)	0.3	0.190	0.4	0.030
October	618(40)	0.1	0.787	0.1	0.762
November	320(21)		Ref		
**Consumes alcohol**					
No	1232(80)	0.2	0.454	-0.4	0.081
Yes	315(20)		Ref		
**Total number of children ever born (parity**										
1 child	338(22)	-0.4	0.245	1.0	0.000
2 children	341(22)	-0.5	0.064	0.4	0.058
3 children	276(18)	-0.3	0.193	0.4	0.115
4 children	593(38)		Ref		

On food items’ consumption, there were no differences between pregnant and non-pregnant women with the exception of ‘other fruits’ (Table 1). Mothers who were currently breastfeeding were more likely to consume dark green leafy vegetables compared to non-breastfeeding mothers, the reverse is true for tinned/powder or fresh milk. Interestingly, with exception of baby formula and cereals, non-breastfeeding children were more likely to consume all the food items compared to their breastfeeding counterparts (Table 1).

### Maternal-child dyad dietary patterns

Table 4 shows four dietary patterns identified by factor analysis with 16 food items among women. These dietary patterns were named according to food item/group factor loadings: “Beverage & sugary based”, “Meat based”, “Indigenous- tuber based” and “Indigenous- grain based” for the mothers. *Beverage and sugary based diet* was characterized by Tea or coffee; Chocolates, sweets, candies, and pastries; Tinned, powdered or fresh milk; and Cheese, yogurt, other milk products. Most of the food items in this group are produced outside of Ghana. The *meat based diet* was characterized by Meats (beef, pork, lamb, chicken); Liver, kidney, heart, other internal organs; and Pumpkins, carrots, yellow or orange squash. The third dietary pattern—*Indigenous- tuber based diet* was largely characterized by potatoes, cassava, or other tubers; other fruits; and fish or shellfish (fresh or dried). Interestingly, the fourth pattern (*indigenous- grain based*) is another indigenous based dietary pattern characterized by Dark green leafy vegetables; Bread, rice, noodles, grains/cereals foods; and Beans, peas, lentils, legumes and nuts. The two indigenous dietary patterns largely depict the kind of foods grown in various locations and staples of the various ethnic groups in the country.

**Table 4:** Extracted dietary patterns and their percentages of explained variance:2008 Ghanaian Demographic and Health Surveys.

Food item	Dietary patterns and the food items loading on each dietary pattern*					
	Mothers (4 dietary patterns)				*Children (2 dietary patterns)*	
	#Beverage & sugary based	#Meat based	#Indigenous- tuber based	#Indigenous- grain based	*#Indigenous*	*#Milk*, *Meat*, *& cereal based*
Bread, rice, noodles, grains/cereals foods	22	-19	-29	61*	*75* ^*^	*14*
Potatoes, cassava, or other tubers	-18	32	65*	-13	*64* ^*^	*3*
Mother had eggs	28	34	7	-13	*32*	*44* ^*^
Meat (beef, pork, lamb, goat,	24	51*	-7	12	*33*	*42* ^*^
Pumpkin, carrots, squash (yel	11	51*	7	6	*30*	*24*
Dark green leafy vegetables	-17	29	11	60*	*64* ^*^	*2*
Mangoes, papayas, other vitamin A foods	-2	36	7	8	*16*	*24*
Other fruits	22	1	54*	16	*67* ^*^	*19*
Liver, kidney, heart, other internal organs	20	55*	-6	4	*22*	*39*
Fish or shellfish (fresh or dried)	18	-39	57*	17	*72* ^*^	*-2*
Beans, peas, lentils, legumes and nuts	1	11	16	55*	*44* ^*^	*9*
Cheese, yogurt, other milk products	44*	29	2	2	*12*	*46* ^*^
Oil, fats, butter, products made from them	25	6	32	37	*63* ^*^	*18*
Tea or coffee	68*	9	1	9	*26*	*41* ^*^
Chocolates, sweets, candies, and pastries	49*	13	14	5	*38*	*43* ^*^
Tinned, powdered or fresh milk	76*	7	3	-2	*5*	*71* ^*^
Other solid-semisolid food	-	-	-	-	*43* ^*^	*11*
Baby formula	-	-	-	-	*-19*	*54* ^*^
Baby cereal	-	-	-	-	*-11*	*60* ^*^
Other porridge/gruel	-	-	-	-	*43* ^*^	*24*
Variance explained	1.91	1.57	1.29	1.29	*3*.*97*	*2*.*55*

Printed values are multiplied by 100 and rounded to the nearest integer. Values greater than 40 are flagged by an ’*’. #Name of extracted dietary pattern

Two dietary patterns emerged for the children using 20 food items: *Indigenous; and Milk*, *Meat*, *& cereal based dietary patterns*. The indigenous pattern for the children is a combination of the two indigenous pattern for their mothers with the addition of “Other solid-semisolid food” and “Other porridge/gruel”. *Milk*, *Meat*, *& cereal based* dietary patterns is characterized by milk and milk based products, tea or coffee, meat and baby cereals. Like the *Beverage and sugary based diet*, most of the food items in this group are produced outside of Ghana.

### Determinants of mother-child dyads dietary patterns

Determinants of mother-child dyads dietary patterns are presented in Tables 2 and 3. Mothers who had household membership of 6, were married, were of Ewe ethnicity, gave birth only once, and mothers who had their interview during the month of September were more likely to practice the ‘beverage and sugary base’ dietary pattern, while women in the lower wealth quintiles were less likely to practice same dietary pattern. The “meat based” dietary pattern was less likely to be practiced by mothers of Ewe and Mole-Dagbani ethnicity but was more likely to be practiced my monoparious mothers. Similarly, mothers of Akan, Ewe, and Guan ethnicities were more likely to practice the “Indigenous- tuber based” dietary pattern. On the “Indigenous- grain based” dietary pattern, mothers of Akan ethnicity and mothers from southern Ghana were less likely to practice the said dietary pattern.

For the children dietary patterns, children who were 12 months or younger, and have mothers within the age brackets of 26–35 years were less likely to practice the indigenous dietary pattern while children whose mothers had primary education were more likely to practice same dietary pattern. Children whose mothers were normal or underweight, belong to households’ with lower wealth quintiles and had no children under five years were less likely to practice the “milk, meat, & cereal based” dietary pattern while children from monoparious mothers and had their interview in September were more likely to practice same dietary pattern.

## Discussion

In this national representative sample, four (‘*Beverage & sugary based’*, *‘Meat based’*, *‘Indigenous- tuber based’ and ‘Indigenous- grain based’*) and two (‘*Indigenous’ and ‘Milk*, *Meat*, *& cereal based’)* dietary patterns respectively emerged for food habits of Ghanaian women and their children (Refer to Table 4). Dietary pattern of children older than one year was similar to their mothers. The similarity in mothers and their children dietary patterns is in consonance with the findings of a similar study in Nigeria [32]. When children are introduced to complementary foods, they are often introduced to family foods. This might be the reason for the similarity in mother child-dyads dietary patterns. It is also important to note that, similar to most impoverished nations, meals in our research settings are monotonous and comprise of a small number of plant base foods [33, 34]. The monotonous nature of diets could also be the reason for the similarity in mother-child dyads’ dietary patterns. One 24-hour recall used in this study is sufficient to approximate routine or customary food intake in this environment due to the monotony of the diet [35].

The results further indicate that ethnicity is a key determinant for all dietary patterns for mothers (Refer to Table 2). Another shared determinant is parity which is common between *Beverage & sugary based* and *Meat based* dietary patterns. These two dietary patterns speak more of social structure, only the elite or those with requisite purchasing power may practice these dietary patterns [36]. In an earlier study in northern Ghana, only the elite or those with requisite purchasing power practiced the *Beverage & sugary based* and *Meat based* dietary patterns [36].

The dietary patterns pointing to social structure is further being buttressed in our study as mothers in the lower wealth quintiles were less likely to practice the *Beverage & sugary based* dietary pattern. The two indigenous dietary patterns speak largely to ethnic staple foods within Ghana. The results indicate that residence of southern Ghana were less likely to consume the *indigenous-grain based* dietary pattern which is typical of northern Ghana.

The determinants for the two dietary patterns for the children (Refer to Table 3) were different as the patterns speak largely to consumption per developmental or physiological stage of children. Children in the breastfeeding category (one year or less) were remarkably less likely to consume the indigenous dietary pattern as compared to their older counterparts. This may be due to younger children starting or being introduced to complementary foods and are not yet on family or household diets.

Aside the physiological status of the children, *Milk*, *Meat*, *& cereal based* dietary pattern shares common determinants (wealth status, parity, month of interview) with the *Beverage & sugary based* dietary pattern of their mothers suggesting that only the elite or those with requisite purchasing power may be able to buy baby formula, meat and commercially processed baby cereals.

It was also interesting to note that children with younger mothers and those with primary education were less likely to practice the indigenous dietary pattern. This finding resonates with an Italian study which indicated that young age and low educational level of mothers were determinants of certain dietary patterns [37]. As mother-child dyad dietary patterns are related [32], it may not be far-fetched why children of younger mothers were less likely to practice the indigenous dietary pattern in our study.

The utilization of a nationally representative sample is one of this study’s strengths. Furthermore, it offers a potential foundation to predict the association between Ghanaian dietary patterns and development of diseases. To the best of our knowledge, this study is the first to characterize the dietary patterns of Ghanaian women and their children at the national level and to look into the socioeconomic and demographic determinants of the dietary patterns.

We are aware that our study has certain limitations. While detailed dietary intake assessment was not initially intended for the Ghana demographic and health survey, only qualitative responses were gathered. However, the robust nature of the principal component analysis accommodated for the qualitative responses. While it is the most recent national data on food intake for women and their children, the current findings are based on an outdated data set (more than a decade old). It is thus conceivable that they may not accurately reflect current intakes and patterns. To validate the current findings and determine whether dietary patterns have altered over the previous fifteen years, more research is required. The complexity of diet can also be captured by the dietary pattern approach, however labeling or naming these patterns may introduce bias on the part of the researchers. The use of these findings could be restricted to settings similar to Ghana.

## Conclusion

Ghanaian mother-child dyads’ dietary patterns were mainly indigenous staple based and meat based. These dietary patterns were significantly influenced by social structure, ethnicity and other vital sociodemographic variables. Additionally, a positive relationship between mother and child dyads’ dietary patterns was found, this suggests that interventions targeted at infant food intake should not be tackled in isolation but that mothers’ food intake should be catered for in such interventions. These findings offer the empirical underpinnings for interventions, suggestions, and policy programs in Ghana that are geared toward women and children. It also acts as a guide for future research and a potential starting point for investigations on the relationship between Ghanaian dietary patterns and disease development. Considering the period in which data for this study was collected, it is likely that these findings may not accurately reflect current intakes and patterns. To validate the current findings, there is an urgent need for a research to determine whether mother-child dyads’ dietary patterns have altered over the past fifteen years.

## Supporting information

S1 Checklist STROBE statement—checklist of items that should be included in reports of cross-sectional studies. (DOCX)

## List of abbreviation

AOR: Adjusted odds ratio
BMI: Body Mass Index
DHS: Demographic and Health Surveys
DP: Dietary Patterns
GDHS: Ghana Demographic and Health Survey
PCA: Principal component analysis
GSS: Ghana Statistical Service
